# Gradient Projection with Approximate L_0_ Norm Minimization for Sparse Reconstruction in Compressed Sensing

**DOI:** 10.3390/s18103373

**Published:** 2018-10-09

**Authors:** Ziran Wei, Jianlin Zhang, Zhiyong Xu, Yongmei Huang, Yong Liu, Xiangsuo Fan

**Affiliations:** 1 Institute of Optics and Electronics, Chinese Academy of Science, Chengdu 610209, China; jearen_wei@163.com (Z.W.); xzy158@163.com (Z.X.); huangym@ioe.ac.cn (Y.H.); ffanxs@163.com (X.F.); 2 School of Optoelectronic Information, University of Electronic Science and Technology of China, Chengdu 610054, China; yongliu@uestc.edu.cn; 3 University of Chinese Academy of Sciences, Beijing 100039, China

**Keywords:** compressed sensing, convex optimization, L_0_ norm, gradient projection, sparse reconstruction

## Abstract

In the reconstruction of sparse signals in compressed sensing, the reconstruction algorithm is required to reconstruct the sparsest form of signal. In order to minimize the objective function, minimal norm algorithm and greedy pursuit algorithm are most commonly used. The minimum L_1_ norm algorithm has very high reconstruction accuracy, but this convex optimization algorithm cannot get the sparsest signal like the minimum L_0_ norm algorithm. However, because the L_0_ norm method is a non-convex problem, it is difficult to get the global optimal solution and the amount of calculation required is huge. In this paper, a new algorithm is proposed to approximate the smooth L_0_ norm from the approximate L_2_ norm. First we set up an approximation function model of the sparse term, then the minimum value of the objective function is solved by the gradient projection, and the weight of the function model of the sparse term in the objective function is adjusted adaptively by the reconstruction error value to reconstruct the sparse signal more accurately. Compared with the pseudo inverse of L_2_ norm and the L_1_ norm algorithm, this new algorithm has a lower reconstruction error in one-dimensional sparse signal reconstruction. In simulation experiments of two-dimensional image signal reconstruction, the new algorithm has shorter image reconstruction time and higher image reconstruction accuracy compared with the usually used greedy algorithm and the minimum norm algorithm.

## 1. Introduction

Compressed sensing theory was put forward in 2006 by Donoho and Candès et al. The main concept is that the sampling and compression process of the signal are completed by one measurement process with a lesser number of measurements than Nyquist sampling, and then the original signal is recovered directly from the measured signal by a corresponding reconstruction algorithm. The transmission and storage costs of signals are saved, and the computational complexity is reduced [1]. The greatest advantage of compressed sensing is that the amount of data obtained by signal measuring is much smaller than that obtained by conventional sampling methods, which breaks through the limitation of sampling frequency in the Nyquist sampling theorem and makes it possible for one to compress and reconstruct high resolution signals. These advantages of compressed sensing enable compressed sensing to be applied in many fields, for example, medical imaging, intelligent monitoring, infrared imaging, object recognition and a lot of military technology fields related to signal sampling.

From the underdetermined sampling, compressed sensing (CS) can recover the sparse discrete signal, which enables compressed sensing to have a wide range of applications [2]. A measured signal y, $y\in C^{m}$, is obtained by the m×n measurement matrix Φ [1,3,4,5], from the original signal, $x\in C^{n}$, m<n. The basic model is as follows:

(1) y=Φx+e,

where e is white Gaussian noise of variance $\delta^{2}$.

CS mainly includes three parts: the sparsity of the signal, the design of the measurement matrix and the recovery algorithm [6]. This paper mainly discusses the recovery algorithm. Specially, for compressed sensing signal reconstruction, there are some excellent algorithms based CS mentioned in [7,8] about recovery of images and wireless image sensor networks.

The signal reconstruction algorithm of compressed sensing is equivalent to the inverse process of signal measurement, that is, the reconstruction of the original signal x through the measurement vector y. And in the process, because the number of equations is far less than the dimension of the original signal, an underdetermined equation must be solved, it is very difficult for direct solving. Therefore, the original signal must be sparse or can be expressed sparsely [4,6,9,10]. Sparse representation is usually represented by the minimum L_0_ norm, which is as the regular expression term in Equation (1). So new expression is obtained as follows:

(2) $$\hat{x}=\arg\underset{x}{\min}{\left\|x\right\|}_{0}\;s.t.\;\Phi x=y,$$

where $\hat{x}$ is an estimate of x.

**Figure 1:**
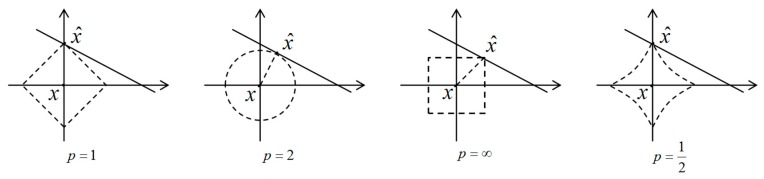
Best approximation of a point in $R^{2}$ by a one-dimensional space using the L_P_ norms for P = 1, 2, ∞, and the L_P_ quasinorm with $p=\frac{1}{2}$.

${\left\|x\right\|}_{0}$ represents the L_0_ norm of x, which represents the total number of non-zero elements in x. The non-convex optimization algorithm by the minimum L_0_ norm method can reconstruct the sparsest expression of the signal, which requires less measurement times. However, it is usually very difficult to solve the minimum L_0_ norm, which is a NP hard problem. For example, if there are K non-zero values in the sparse signal with a length of N, and there are $C_{N}^{K}$ forms of permutation, which makes easy for the algorithm to fall into a local optimum. To find the optimal permutation closest to the original signal, the computational complexity is very high and it is very high time-consuming. However, with the convex optimization algorithm of L_1_ or L_2_ norm is easy to get the global optimal value and can be guaranteed theoretically, but it does not ensured to be the sparsest of reconstructed signals, which is described in detail later in Figure 1. Moreover, the amount of computation is still large, which is only suitable for small scale signals. For efficient sparse signal reconstruction, the L_0_ norm problem is often circumvented. For example, the sub-optimal algorithm represented by the minimum L_1_ norm method [11,12] and the greedy algorithm represented by the orthogonal matching pursuit algorithm [13] are usually chosen to solve the problem, but the convergence and stability of the two algorithms need further theoretical guarantees.

Instead of minimizing the L_0_ norm in Equation (2), adopting the L_1_ norm and Equation (2) becomes:

(3) $$\hat{x}=\arg\underset{x}{\min}{\left\|x\right\|}_{1}\;s.t.\;\Phi x=y.$$

The presence of the L_1_ term encourages small components of x to shrink to zero and promotes sparse solutions [12,14]. Theoretically, Equations (2) and (3) are approximately equivalent, but Equation (2) can obtain a sparser solution. Equation (3) is a convex optimal problem which can be solved by linear programming. Actually, there is a certain error between the reconstructed signal and the original signal. The residual value between them can be used to assess the approximation and accuracy of reconstructed signal, so Equation (3) can usually be expressed as:

(4) $$\underset{x}{\min}{\left\|x\right\|}_{1}\;s.t.\;{\left\|\Phi x-y\right\|}_{2}^{2}\leq \varepsilon ,$$

and:

(5) $$\underset{x}{\min}{\left\|\Phi x-y\right\|}_{2}^{2}\;s.t.\;{\left\|x\right\|}_{1}\leq t,$$

where ε and t are real parameters with nonnegative values.

Equation (4) is a quadratically constrained linear program (QCLP) problem, whereas Equation (5) is a quadratic program (QP) [15]. Both Equations (4) and (5) are convex optimization problem, they easily reach the global optimal value, but they cannot guarantee the signal to be the sparsest. In addition, because L_1_ norm cannot be differentiated at zero, the L_1_ norm is non-analytic at zero and usually has a large amount of computation. For non-convex L_0_ norm optimization, the theoretical guarantee of the uniqueness of the global optimal solution is very weak, but the L_0_ norm non-convex reconstruction performs better than the L_1_ norm reconstruction at low sampling rates [16]. In order to utilize the advantages of the L_0_ norm and L_1_ norm reconstruction model, simultaneously reducing the number of measurements and the complexity of computation, the compromising minimum L_P_ (0 < P < 1) norm algorithm is proposed in [17]. That is:

(6) $$\underset{x}{\min}{\left\|\Phi x-y\right\|}_{2}^{2}\;s.t.\;{\left\|x\right\|}_{P}\leq t.$$

There are notably different properties of L_P_ norms for different values of P. To illustrate this, in Figure 1 the unit sphere, i.e., $\left\{x:{\left\|x\right\|}_{P}=1\right\}$, is induced by each of these norms in $R^{2}$. We use a L_P_ norm to measure the approximation error and our task is to find $\hat{x}\in A$ that minimizes ${\left\|x-\hat{x}\right\|}_{P}$ [18]. As shown in Figure 1, to find the closest point in A to x by L_P_ norm, we grow the L_P_ sphere centered on x until it intersects with A. There will be the point $\hat{x}\in A$ that is closest to x for the corresponding L_P_ norms. Through observation, we can know that a larger P tends to spread out the error more evenly among the two coefficients of two coordinate axes, while a smaller P leads to an error that is more unevenly distributed and tends to be sparser [18].

The above analysis is also applicable to higher dimensional situations, which plays an important role in CS theory. Reference [17] theoretically analyzes the feasibility of using L_P_ norm instead of L_0_ norm. When the Restricted Isometric Property (RIP) condition [4,19] of the measurement matrix is weakened, the number of measurements needed for accurate reconstruction of the minimum L_P_ norm method is greatly reduced compared with the minimum L_1_ norm method. Reference [20] further illustrates the necessary conditions of the parameter P and the constraint isometric constant (RIC) for the accurate reconstruction.

The Iterative Reweighted algorithm is proposed in [21,22] to solve the minimum L_P_ norm problem. Some researchers use the method of dynamically shrinking parameter P (P asymptotically approximating to zero) to solve the minimum L_P_ norm problem in the iterative process of the algorithm, which is described in detail in [23]. Other researchers fixed P to a value of 0 to 1, for example, in the L_1/2_ regularization algorithm [24]. Based on this idea of approximate L_0_ norm minimization, this paper proposes a new algorithm from L_2_ norm to approximate L_0_ norm by the method of gradient projection. The non-analytic problem of the L_1_ norm at zero is avoided, and the advantages of the convex optimization and non-convex optimization are also synthesized.

The remainder of this paper is organized as follows: in Section 2, we present a theoretical analysis of the new algorithm and introduce the basic algorithm framework. In Section 3, we introduce the specific algorithm process of L_2_ norm to approximate L_0_ norm using the method of gradient projection. In Section 4, simulation experiments are carried out to compare the new proposed algorithm with the traditional classic algorithms for the reconstruction of one-dimensional and two-dimensional signals. Finally, conclusions are drawn in Section 5.

## 2. Formulation from L_2_ Norm to Approximate L_0_ Norm

Our novel proposed algorithm approximates L_0_ norm from L_2_ norm based on our insight of the relationship between the L_2_ norm and L_0_ norm. In the algorithm, we do not set specific parameter P in L_P_ norm. To approximate L_0_ norm, we first define an approximation function model. In the iterative process of new algorithm, the L_0_ norm is gradually approximated by changing the value of a modulation parameter in the function model. Thus, we can approximate the global optimal solution and the sparsest solution with greater probability and efficiency.

In Equation (1), x is the original signal, but generally the original signal maybe is not necessarily sparse. Therefore, the compressive sensing measurement and reconstruction of signal (Equations (1)−(6)) cannot perform well, and the original signal x must be transformed into some sparse domain i.e., x=Ψs, Ψ is a sparse transformation base, and s is a sparse representation signal. Thus y=Θs, Θ=ΦΨ, Θ is called the sensing matrix.

**Figure 2:**
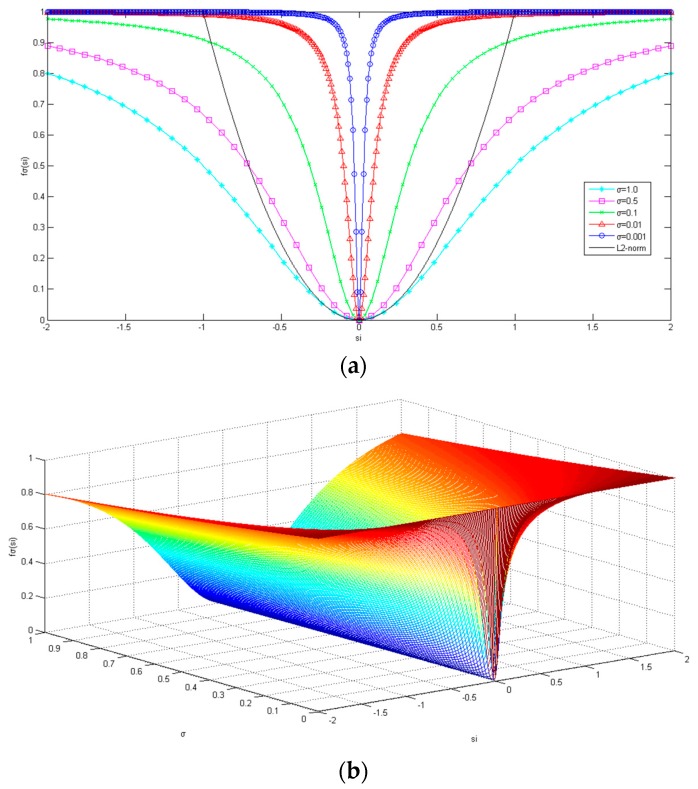
(**a**) A 1D example of $f_{\sigma }\left(s_{i}\right)$ with different σ values. The L_2_ norm of the black solid line is included for comparison. (**b**) A 2D example surface plot of $f_{\sigma }\left(s_{i}\right)$.

To approximate L_0_ norm, an approximation function model is first defined as:

(7) $$f_{\sigma }\left(s\right)=\frac{{\left\|s\right\|}_{2}^{2}}{{\left\|s\right\|}_{2}^{2}+\sigma }\left(f_{\sigma }\left(s_{i}\right)=\frac{{\left|s_{i}\right|}^{2}}{{\left|s_{i}\right|}^{2}+\sigma }\right),$$

where $s_{i}$ is the ith element in the sparse signal s, and σ is the modulation parameter approximating the L_0_ norm, $f_{\sigma }\left(s_{i}\right)$ is the function value of the element $s_{i}$ in the sparse signal. The relation diagram of function $f_{\sigma }\left(s_{i}\right)$ and $s_{i}$ and σ is shown in Figure 2.

As shown in Figure 2, when σ is large, $f_{\sigma }\left(s_{i}\right)$ is approximately the L_2_ norm of signal s when $s_{i}$ is close to zero, as shown in the black solid line. When the value of σ decreases gradually, the $f_{\sigma }\left(s_{i}\right)$ gradually approaches the L_0_ norm of signal s, as shown in the middle curve of Figure 2a, that is:

(8) $$f_{\sigma }\left(s_{i}\right)\approx \{\begin{array}{c} 1,\;\left|s_{i}\right|\gg \sigma \\ 0,\;\left|s_{i}\right|\ll \sigma \end{array}.$$

That is, when $s_{i}$ is large, it is 1, and when $s_{i}$ is small, it is 0. For the summation of $f_{\sigma }\left(s_{i}\right)$, we can get:

(9) $$F_{\sigma }\left(s\right)=\sum_{i=1}^{n}f_{\sigma }\left(s_{i}\right),$$

where n is the length of the sparse signal vector s. Obviously Equation (9) is equivalent to approximately calculating the number of non-zero items of sparse vector s, which is equivalent to get the L_0_ norm of sparse vector s when the σ sigma is very small, i.e., the optimization problem of the L_0_ norm can be nearly as ${\left\|s\right\|}_{0}\approx F_{\sigma }\left(s\right)$. As shown in Figure 2, since the functional curve approximating L_0_ norm is smooth and derivable, it is also convenient to get the derivative of $F_{\sigma }\left(s\right)$ and the gradient of the objective function. Thus, minimizing the discontinuous function ${\left\|s\right\|}_{0}$ is transformed into minimizing the continuous function $F_{\sigma }\left(s\right)$. Thus, Equation (2) is transformed into:

$$\hat{x}=\arg\underset{x}{\min}F_{\sigma }\left(s\right)\;s.t.\;\Theta s=y,$$

that is:

(10) $$\hat{x}=\arg\underset{x}{\min}F_{\sigma }\left(s\right)\;s.t.\;{\left\|\Theta s-y\right\|}_{2}^{2}\leq \varepsilon .$$

For the inverse problem of signal reconstruction, the prior of sparse representation is as a regularization term (the penalty function, $F_{\sigma }\left(s\right)$), so in order to obtain the optimal solution of the signal reconstruction, the constrained optimization of the approximation signal item should be considered. Based on the model Equation (10), Lagrangian method is applied to this constrained optimization problem, we add the residual of the reconstructed signal, and the compressed sensing signal reconstruction model with approximation L_0_ norm sparse representation is formed as:

(11) $$\arg\underset{s}{\min}\left\{J\left(s\right)=\lambda F_{\sigma }\left(s\right)+\frac{1}{2}{\left\|\Theta s-y\right\|}_{2}^{2}\right\}.$$

In Equation (11) J(s) is the objective function we need to minimize. The goal of this algorithm is to seek the estimated value of sparse vector s which minimizes the objective function, where λ is the weight parameter used to adjust the weight of the sparse representation. Constant 1/2 of the reconstructed signal residual term is convenient to calculate the derivation of objective function. The sparse representation item in Equation (11) constrains the sparsity of signal s, and the reconstructed signal residual term is the minimal difference between the reconstructed signal measurement value and the actual measurement value, which can be considered as a global optimization for the whole signal. In order to find the minimum value of the target function J(s), the parameter σ in $F_{\sigma }\left(s\right)$ is gradually reduced to make $F_{\sigma }\left(s\right)$ approximate to the L_0_ norm, so it is necessary to reduce the value of the parameter σ in the iterative process of the new proposed algorithm.

For general compress sensing signal reconstruction, the iterations of non-convex algorithm are usually prone to trapping at suboptimal local minima, for the reason that there are always combinatorial numbers of solutions for sparse signal. However, by applying $F_{\sigma }\left(s\right)$ in Equation (11), the problem of local minima can be implicitly avoided by solving it with a large initial σ beginning, such that the penalty function $F_{\sigma }\left(s\right)$ is initially nearly convex as ${\left|x\right|}^{2}$ (see Figure 2). As the iterations proceed and the details of the signal need to be reconstructed, the penalty function $F_{\sigma }\left(s\right)$ becomes less convex when σ has shrunk to the small, and is approximate to the L_0_ norm, but the risk of local minima and instability the solution falls into is ameliorated by the fact that the solution is already in the neighborhood of a desirable attraction basin of the global optimum. Thus, the implicit noise level (or modeling error between the reconstructed signal and the original signal) is now substantially less.

In this paper, the iterative gradient projection method is used to seek the minimum value of the objective function J(s). The gradient ΔJ(s) of the objective function is obtained by deriving it with respect to s in (11):

(12) $$\Delta J\left(s\right)=d=\left(\Theta^{T}\left(\Theta s-y\right)\right)+2\lambda {\left[\frac{s_{1}\sigma }{{\left(s_{1}{}^{2}+\sigma \right)}^{2}},\ldots ,\frac{s_{n}\sigma }{{\left(s_{n}{}^{2}+\sigma \right)}^{2}}\right]}^{T}.$$

Based on gradient projection, the basic algorithm framework of approximation minimum L_0_ norm algorithm is as Algorithm 1.

**Algorithm 1.** Approximation Minimum L_0_ Norm.
Input: the sensor matrix Θ, the measured value y, and the sparse representation s of the original signal.Initialization: ${\hat{s}}^{\left(0\right)}=\Theta^{⊥}y$, $\Theta^{⊥}$ is the pseudoinverse matrix of Θ, $\Theta^{⊥}={\left(\Theta^{T}\Theta \right)}^{-1}\Theta^{T}$. Decreasing sequence of parameter σ, $\sigma =\left[\sigma_{0},{\alpha \sigma }_{0},\alpha^{2}\sigma_{0},\ldots ,\alpha^{k}\sigma_{0}\right]$. Weight parameter λ and gradient descent step length γ.The gradient projection iteration number k:$\sigma =\sigma_{k}$, $\hat{s}={\hat{s}}^{\left(k-1\right)}$.The gradient descent direction:$$d=\left(\Theta^{T}\left(\Theta s-y\right)\right)+2\lambda {\left[\frac{s_{1}\sigma }{{\left(s_{1}{}^{2}+\sigma \right)}^{2}},\ldots ,\frac{s_{n}\sigma }{{\left(s_{n}{}^{2}+\sigma \right)}^{2}}\right]}^{T}$$.Update gradient direction: $\hat{s}=\hat{s}-\gamma d$.Constrained orthogonal projection: $\hat{s}=\hat{s}-\Theta^{⊥}\left(\Theta \hat{s}-y\right)$.${\hat{s}}^{\left(k\right)}=\hat{s}$, k = k + 1.If satisfy the stop iteration condition which is listed in Section 3, end the loop: the output $s^{\left(k\right)}$ is the final sparse signal s.


It is worth noting that, in the process of this algorithm, the ${\left\|\Theta s-y\right\|}_{2}^{2}$ is getting smaller and smaller with the iteration, and the decrease of σ makes the regular penalty term $F_{\sigma }\left(s\right)$ more and more close to the L_0_ norm. In order to reconstruct the original signal more accurately, we can make the weight of the error ${\left\|\Theta s-y\right\|}_{2}^{2}$ increase gradually, and then the regular penalty term $F_{\sigma }\left(s\right)$ is relatively reduced. Therefore, the value of the weight lambda can be adaptively adjusted in the iterative process according to the size of the error ${\left\|\Theta s-y\right\|}_{2}^{2}$, which can be set into a descending sequence that is positively correlation with the value of the ${\left\|\Theta s-y\right\|}_{2}^{2}$.

From Figure 2a,b, it is known that with the gradual decrease of σ, $F_{\sigma }\left(s\right)$ is increasingly approaching the smooth approximation of the L_0_ norm, which gradually constrains the sparsity of the reconstructed sparse signal and gradually approaches the global optimal solution in greater efficiency. Besides, subsequent iterations of the new algorithm begin to reflect the correct coarse shape, σ can be gradually reduced to allow the recovery of more detailed, fine structures. Thus the proposed algorithm reduces the complexity of the algorithm and improves the accuracy of the signal reconstruction. It is more conducive to signal reconstruction of compressed sensing. The next section will introduce the specific implementation of gradient projection in the new approximating L_0_ norm algorithm in detail.

## 3. The Gradient Projection Implementation in the Approximating L_0_ Norm Algorithm

In this section, we discuss GP (gradient projection) techniques for solving Equation (11). In order to minimize the objective function in Equation (11), as in [25], we split the variable s into the positive and negative parts. Therefore, we introduce vector u and v to get the following substitution:

(13) s=u−v, u≥0, v≥0,

where $u_{i}={\left(s_{i}\right)}_{+}$ and $v_{i}={\left(-s_{i}\right)}_{+}$ for all i=1,2,…,n, n is the length of the vector s. ${\left(s\right)}_{+}$ is the positive-part operator defined as ${\left(s\right)}_{+}=\max\left\{0,s\right\}$, so Equation (11) can be rewritten as the following bound-constrained quadratic program (BCQP):

(14) $$\arg\underset{s=u-v}{\min}\left\{J\left(s\right)={\lambda F}_{\sigma }\left(u-v\right)+\frac{1}{2}{\left\|\Theta \left(u-v\right)-y\right\|}_{2}^{2}\right\},$$

where, u≥0, v≥0. Further Equation (14) can be rewritten as:

(15) $$\arg\underset{z}{\min}\left\{J\left(z\right)={\lambda F}_{\sigma }\left(z\right)+c^{T}z+\frac{1}{2}z^{T}\mathrm{Az}\right\},$$

where, $z=\left[\begin{array}{c} u\\ v \end{array}\right]\geq 0$, $b=\Theta^{T}y$, $c=\left[\begin{array}{c} -b\\ b \end{array}\right]$, and:

(16) $$A=\left[\begin{array}{cc} \Theta^{T}\Theta & -\Theta^{T}\Theta \\ \Theta^{T}\Theta & \Theta^{T}\Theta \end{array}\right].$$

In order to adjust the value of the sparsity term weight λ adaptively according to the error ${\left\|\Theta s-y\right\|}_{2}^{2}$, we set λ to:

(17) $$\lambda =0.1{\left\|\Theta s-y\right\|}_{\infty }.$$

We can observe that the dimension of Equation (15) is twice that of the original problem (11): $s\in R^{n}$, while $z\in R^{2n}$. However, this increase in dimension has only a minor impact on matrix operations, which can be performed more economically than its size suggests, by applying the particular structure (16) [15]. For a given $z^{T}=\left[u^{T}v^{T}\right]$, we have:

(18) $$z^{T}\mathrm{Az}={\left(u-v\right)}^{T}\Theta^{T}\Theta \left(u-v\right)={\left\|\Theta (u-v)\right\|}_{2}^{2},$$

indicating that using only a single multiplication by Θ can calculate the quantity. Since $J\left(z\right)={\lambda F}_{\sigma }\left(z\right)+c^{T}z+\frac{1}{2}z^{T}\mathrm{Az}$, in the same way that evaluation of J(z) also needs only one multiplication by Θ.

In order to solve the objective function of Equation (15), we introduce the scalar parameter $\mu^{\left(k\right)}>0$ and update z from iterate $z^{\left(k\right)}$ to iterate $z^{\left(k+1\right)}$ as follows:

(19) $$z^{\left(k+1\right)}={\left(z^{\left(k\right)}-\mu^{\left(k\right)}\nabla J\left(z^{\left(k\right)}\right)\right)}_{+}.$$

For each iteration of $z^{\left(k\right)}$, we search along the negative gradient direction $-\nabla J\left(z^{\left(k\right)}\right)$, projecting onto the non negative orthant and conducting a backtracking line search until a sufficient decrease is attained in $J\left(z^{\left(k\right)}\right)$. (Bertsekas [26] refers to this strategy as “Armijo rule along the projection arc.”) There is an initial hypothesis that the technique would yield the exact smller value of $J\left(z^{\left(k\right)}\right)$ along this direction if there are no new bounds to be encountered [15]. Specifically, the vector is defined as:

$$g_{i}{}^{\left(k\right)}=\{\begin{array}{c} {\left(\nabla J\left(z^{\left(k\right)}\right)\right)}_{i},\;z_{i}{}^{\left(k\right)}>0\;\mathrm{or}\;{\left(\nabla J\left(z^{\left(k\right)}\right)\right)}_{i}<0\\ 0,\;\mathrm{otherwise}. \end{array},$$

where i=1, 2,…, 2n, 2n is the number of elements in z vector, and we initialize it by the following equation.

$$\mu_{0}=\arg\underset{\mu }{\min}J\left(z^{\left(k\right)}-{\mu g}^{\left(k\right)}\right),$$

which can be computed by:

(20) $$\mu_{0}=\frac{{\left(g^{\left(k\right)}\right)}^{T}g^{\left(k\right)}}{\left(g^{\left(k\right)}\right)A^{T}g^{\left(k\right)}}.$$

To prevent the value of $\mu_{0}$ from being too large or too small, we usually limit $\mu_{0}$ to an interval $\left[\mu_{\min},\mu_{\max}\right]$, where $0<\mu_{\min}<\mu_{\max}$. That is, $\mu_{0}=\mathrm{mid}\left(\mu_{\min},\mu_{\max},\mu_{0}\right)$, the operator mid(a,b,c) are defined as the middle value of three scalar arguments. When $\left(g^{\left(k\right)}\right)A^{T}g^{\left(k\right)}=0$, $\mu_{0}=\mu_{\max}$. This method produces a more acceptable value of $\mu_{0}$ than the $\mu_{0}$ computed only by Equation (20) along the direction $-\nabla J\left(z^{\left(k\right)}\right)$.

The implementation steps of the gradient projection algorithm approximating the L_0_ norm are shown in Algorithm 2.

**Algorithm 2.** The Gradient Projection Approximating the L_0_ Norm.
Initialization: As shown in Section 2, $s^{\left(0\right)}=\Theta^{⊥}y$, $m^{\left(0\right)}={\left(s^{\left(0\right)}\right)}_{+},\;n^{\left(0\right)}={\left(-s^{\left(0\right)}\right)}_{+},\;z^{\left(0\right)}=\left[\begin{array}{c} m^{\left(0\right)}\\ n^{\left(0\right)} \end{array}\right]$; decreasing parameter of $\mu_{0}$, α∈(0,1), and gradient descent step parameter, $\gamma \in \left(0,\frac{1}{2}\right)$; the number of iterations k, k = 0.Calculate the value of $\mu_{0}$ by Equation (20), and the value of $\mu_{0}$ is determined by $\mathrm{mid}\left(\mu_{\min},\mu_{\max},\mu_{0}\right)$.The line search of backtracking: Choose the first number in the sequence $\mu_{0},{\beta \mu }_{0},\beta^{2}\mu_{0},\ldots$ as $\mu^{\left(k\right)}$, such that (21)$$J\left({\left(z^{\left(k\right)}-\mu^{\left(k\right)}\nabla J\left(z^{\left(k\right)}\right)\right)}_{+}\right)\leq J\left(z^{\left(k\right)}\right)-\gamma J{\left(z^{\left(k\right)}\right)}^{T}\left(z^{\left(k\right)}-{\left(z^{\left(k\right)}-\mu^{\left(k\right)}\nabla J\left(z^{\left(k\right)}\right)\right)}_{+}\right),$$
and set $z^{\left(k+1\right)}={\left(z^{\left(k\right)}-\mu^{\left(k\right)}\nabla J\left(z^{\left(k\right)}\right)\right)}_{+}$.When the algorithm performs convergence and satisfies the termination condition (22)$$\frac{{\left\|\nabla J(z^{\left(k\right)})\right\|}_{2}}{{\left\|z^{\left(k\right)}\right\|}_{2}}\leq \mathrm{tolA},$$
the iteration is stopped, and $z^{\left(k+1\right)}$ is the approximate solution of z; otherwise set k = k + 1 and return to 2 and 3, until the termination condition is satisfied.


$\nabla J\left(z^{\left(k\right)}\right)$ is the gradient of J(z) at $z^{\left(k\right)}$ for the kth iteration, and $z^{\left(k\right)}$ is the value of z at the kth iteration, tolA is a constant of lower limit and the default value is 0.01. As the new proposed algorithm approximates to L_0_ norm minimization by the way of gradient projection, we refer to it as L0GP algorithm.

## 4. Experimental Analysis and Discussion

In the section, to verify the effectiveness of the proposed L0GP algorithm, simulation experiments are carried on by Matlab to reconstruct one-dimensional signal and two-dimensional image signals. For one-dimensional signal reconstruction experiments, we compare our algorithm L0GP with the classic L1-Regularized Least Squares (L1_LS) algorithm of L_1_ norm [11] such as Equation (3), and with the algorithm of minimum L_2_ norm, since the new proposed L0GP algorithm iteratively approximate L_0_ norm from L_2_ norm.

MSE, the mean square error is used to measure the reconstruction accuracy of one dimensional sparse signal. The definition is as follows:

(23) $$\mathrm{MSE}=\left(1/n\right){\left\|\hat{x}-x\right\|}_{2}^{2},$$

where $\hat{x}$ is the estimate of x.

In the experiments, we take full account of a typical signal reconstruction of CS (similar to [11]), and our goal is to reconstruct an original sparse signal with a length of n and sparsity of k through a measured signal of a length of m, in which m < n. The signal are measured in space domain by matrix Φ, which is a Gauss random matrix of m×n size, and each element of the matrix is subject to a Gauss distribution with a mean value of 0 and a variance of 1/m. In this experiment, n = 4096, m = 1024, sparsity k = 220, which is the original sparse signal with a length of 4096, contains 220 non-zero values of a random distribution of +1, 0 or −1, and the observation y is generated by Equation (1). In the L0GP algorithm, the initial value of parameter λ is chosen as suggested in [11]

(24) $$\lambda_{0}=0.1{\left\|\Phi^{T}y\right\|}_{\infty }.$$

**Figure 3:**
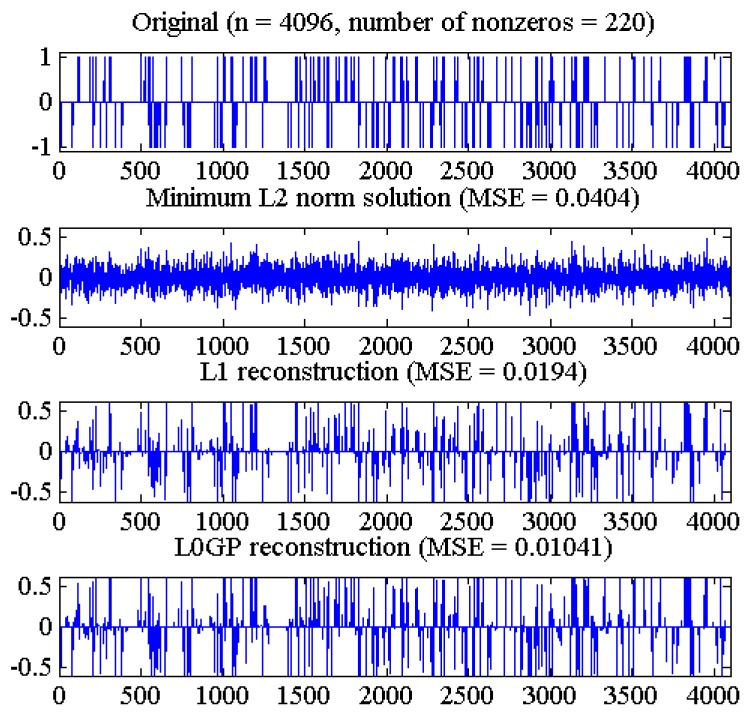
From top to bottom: original signal, reconstruction obtained by the minimum L_0_ norm solution, L1_LS algorithm and the new proposed algorithm.

Notice that for $\lambda \geq 0.1{\left\|\Phi^{T}y\right\|}_{\infty }$, the unique minimum of Equation (3) is the zero vector [11,27], and parameters are set as σ=1, α=0.5, β=0.4, γ=0.2. The values of these parameters are the relatively optimal results of a large number of experiments after we tried different parameters values. In the process of experiments adjusting parameters, we think the performance of our method is not particularly sensitive to these choices, as long as these parameters are in the certain interval. The minimum L_0_ norm solution is given by $\hat{x}=\Phi^{T}{\left({\Phi \Phi }^{T}\right)}^{-1}y$, that is, $\hat{x}$ is the pseudo inverse solution of the undetermined system y=Φx. The reconstruction results of one dimensional signal are shown in Figure 3.

As shown in Figure 3, the L_2_ norm pseudo-inverse method can hardly recover the original signal, and the L_1_ norm algorithm is better than the pseudo-inverse method. The L0GP algorithm is superior to the L_1_ norm and L_2_ norm algorithms, and has smaller reconstruction error and higher reconstruction accuracy.

**Figure 4:**
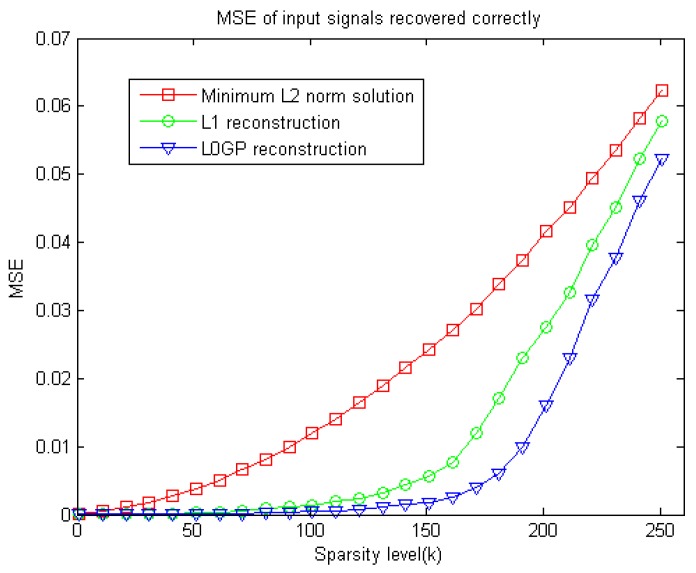
MSE of sparse signal with different sparsity level recovered correctly by the three different reconstruction methods.

Since both the original sparse signal and the measurement matrix are randomly generated, the experiments’ results may be random. To assess these algorithms objectively, five experiments have been done for sparse signal reconstruction with the same sparsity, and then the average of their results is taken as the evaluation. This experiment takes n = 512, m = 128, and obtains the correlation between the reconstruction error of the sparse signal and the signal sparsity K among the three different methods, just as curve shown in the Figure 4.

It is known from Figure 4, that the image reconstruction error of L0GP algorithm is less than the L_1_ norm algorithm and the L_2_ norm pseudo-inverse algorithm in the case of different sparsity. From the above two experiments, it can be seen that the new algorithm shows advantages from convex optimization to non-convex optimization and performs very well for the reconstruction of one dimensional sparse signals.

In order to further prove the good performance of L0GP algorithm, we furtherly do experiments on the reconstruction of non-sparse two-dimensional image signals. These experiments compared the new algorithm with other algorithms, such as Subspace Pursuit (SP) [28], Iterative Reweighted Least Squares (IRLS) [29], Orthogonal Matching Pursuit (OMP) [13], Regularized Orthogonal Matching Pursuit (ROMP) [30], Generalized Orthogonal Matching Pursuit (GOMP) [31], L1-Basis Pursuit (L1_BP) [12] and L1_LS [11].

The size of the experimental image is 512×512. If the two-dimensional image signal is simply arranged in a single column signal of one dimension, the length of the signal would be extremely large and also it will cost a lot of time for reconstruction. Therefore, our approach is to measure and sample the column signal (512×1) and the row signal (1×512) of the image in space domain respectively, which is equivalent to the reconstruction of the column signal or the row signal of the image, so the reconstruction time of the whole image also demonstrates the efficiency of the reconstruction algorithms. Since the two-dimensional image signal is a non-sparse signal in space domain, we need to transform it into the sparse representation domain by wavelet [17]. In this paper, we use discrete wavelet sparse transform (DWT) as the sparse basis, that is, the signal x are represented as s in sparse domain, x=Ψs, Ψ is discrete wavelet basis.

In order to evaluate the accuracy of the reconstructed image, the peak signal to noise ratio (PSNR) and structural similarity (SSIM) are used as the evaluation index of the image, which are defined as follows:

(25) $$\mathrm{PSNR}\left(\mathrm{dB}\right)=20\log\frac{255}{\frac{1}{\mathrm{mn}}\sum_{i}\sum_{j}{\left(\hat{x}\left(i,j\right)-x\left(i,j\right)\right)}^{2}},$$

(26) $$\mathrm{SSIM}=\frac{\left(2\mu_{X}\mu_{\hat{x}}+c_{1}\right)\left(2\sigma_{x\hat{x}}+c_{2}\right)}{\left(\mu_{x}^{2}+\mu_{\hat{x}}^{2}+c_{1}\right)\left(\sigma_{x}^{2}+\sigma_{\hat{x}}^{2}+c_{2}\right)},$$

where $\mu_{X}$ and $\mu_{\hat{x}}$ are the mean values of x and $\hat{x}$ respectively. $\sigma_{x}^{2}$, $\sigma_{\hat{x}}^{2}$ and $\sigma_{x\hat{x}}$ are the variance or covariance values of x and $\hat{x}$ respectively. $c_{1}$ and $c_{2}$ are the constants that maintain stability, $c_{1}={\left(k_{1}L\right)}^{2}$, $c_{2}={\left(k_{2}L\right)}^{2}$. L is the dynamic range of pixel values, $k_{1}=0.01$, $k_{2}=0.03$.

**Figure 5:**
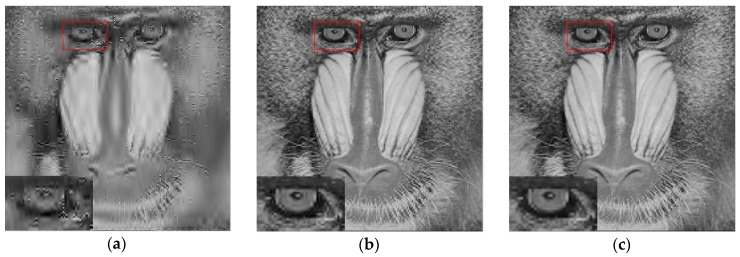

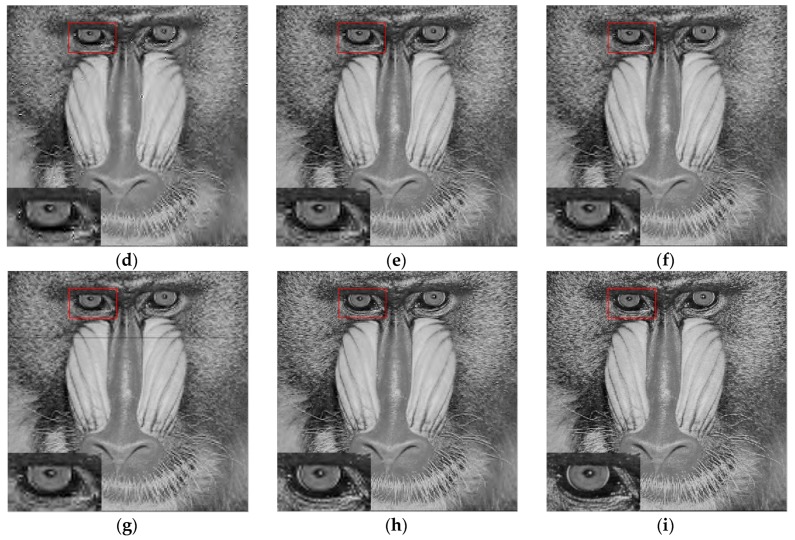
Reconstruction results of different arithmetic of (**a**) SP, (**b**) IRLS, (**c**) OMP, (**d**) ROMP, (**e**) GOMP, (**f**) L1_BP, (**g**) L1_LS, (**h**) L0GP, (**i**) Original Mandrill image.

For the Mandrill image with a size of 512×512 (n = 512×512), we reconstruct the image by m times sampling of the image, m = 28900 (m=170×170), and the sampling rate is about m/n = 1/9. The results of the experiment are shown in Figure 5.

Then, PSNR, SSIM and the time of image reconstruction for the different algorithms are statistically obtained, and the results are shown in the following table.

From Figure 5 and Table 1, we can find that under the sampling rate of 1/9, compared with the suboptimal algorithm of the least norm and the greedy algorithm of the matching pursuit, the PSNR and SSIM of reconstructed image by the new proposed algorithm have been improved to a certain extent, and have higher image reconstruction accuracy. By zooming in the eye area of the mandrill marked by red box, it can be seen that the details of the reconstructed image become clearer and the picture quality is better, and the sharpness of image edge is well enhanced and the noise becomes less.

Because the time of the image reconstruction of the greedy algorithm of the matching pursuit class depends on the number of iterations or the terminating conditions, although the image reconstruction time of ROMP algorithm is very short, the image reconstruction accuracy of ROMP is much worse than that of L0GP. Moreover, when the number of iterations reaches a certain number, the image reconstruction accuracy of ROMP will not increase obviously with the number of iterations, instead, it will consume a lot of time and computation. Owning to fact that the L0GP can quickly approximate the global optimal solution with bigger probability, the reconstruction time is greatly reduced much more than the traditional iterative least square algorithm (IRLS) and the L_1_ norm algorithm. Comparing L0GP with the traditional iterative least square algorithm (IRLS) and L_1_ norm algorithm, the reconstruction time is greatly shortened and the image reconstruction accuracy is improved, so the advantage of this algorithm is evident.

**Figure 6:**
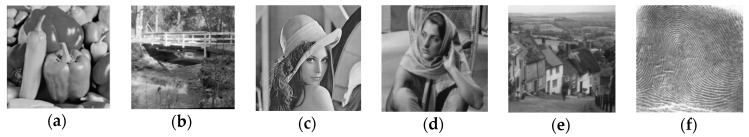
Different representative images of (**a**) Peppers, (**b**) Bridge, (**c**) Lena, (**d**) Barbara, (**e**) Goldhill, (**f**) Fingerprint.

In order to prove the validity and generality of the L0GP algorithm for various image reconstruction, we choose six representative images, including nature scenes, persons, animals, detailed and texture images, as shown in Figure 6. At the same sampling rate of 1/9, these images were reconstructed. The quantitative results of the experiment are shown in Table 2.

Analyzing the data in Table 2, the proposed algorithm has a good performance in the reconstruction of various types of images, and has a good advantage over the other seven algorithms. It shows that the new algorithm has a strong generality and adaptability for all kinds of images.

**Figure 7:**
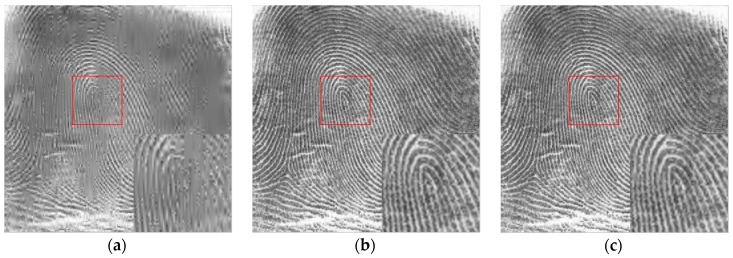

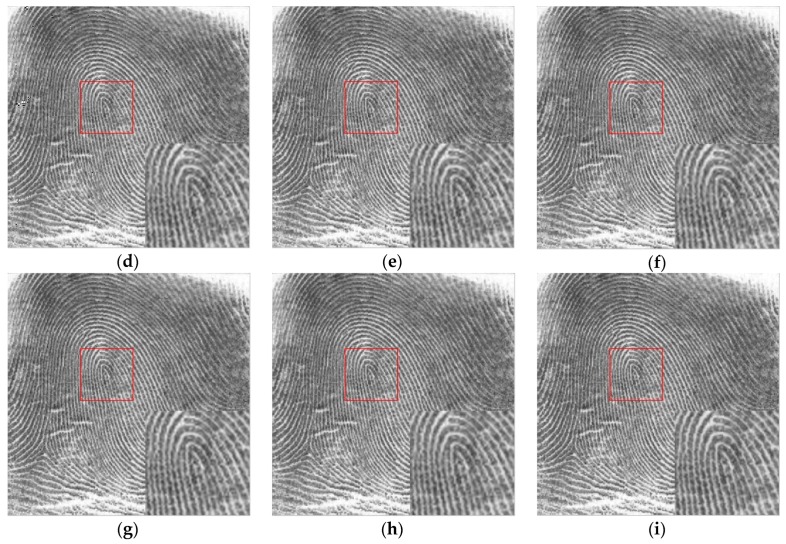
Reconstruction results of different algorithms of (**a**) SP, (**b**) IRLS, (**c**) OMP, (**d**) ROMP, (**e**) GOMP, (**f**) L1_BP, (**g**) L1_LS, (**h**) L0GP, (**i**) Original Fingerprint image.

For the Fingerprint image with a size of 512×512 (n = 512×512), we increase the number of sampling on the image and do the simulation experiment again. We reconstruct the image by m times sampling of image, m = 65536 (m=256×256), i.e., the sampling rate is about m/n = 1/4. The results of the experiment are shown in Figure 7.

As shown in Figure 7 and Table 3, the texture and details of the Fingerprint image are well reconstructed. Clearly, the proposed algorithm show better performance at reconstructing fine structures. Moreover, the blur is obviously suppressed, meanwhile the new algorithm reconstructs more rich information than the others, and the reconstructed image has the highest PSNR and SSIM in the quantitative assessment indexes.

For the large amount of data like image and the reconstruction of high sampling rate signal, the greedy algorithm of the OMP class has weak theoretical guarantee. From the data statistics above Figure 7 and Table 3, the accuracy and time of the reconstruction cannot reach the ideal at the same time, and the accurate reconstruction of the signal has some randomness. Therefore, the greedy algorithm for OMP class often considers the time cost on the basis of satisfying the reconstruction accuracy, and takes a compromise between the reconstruction time and the reconstruction accuracy. Similarly, when the sampling rate is 1/4, different types of images are reconstructed, and statistical data are obtained as shown in Table 4.

Based on the data statistics of the above experiments, with increasing signal sampling rate, more information of the original signal is obtained from sampling, so the advantages of the new proposed algorithm have been weakened slightly. Although the performance of the new proposed L0GP algorithm is not the best in the reconstruction time, the reconstruction time is also very short and the accuracy of the reconstructed signal is the highest among the eight algorithms. The experimental simulation results also further verify the effectiveness of the algorithm theory analysis in Section 2 and Section 3.

## 5. Conclusions

In this paper, a gradient projection algorithm approximating the minimum L0 norm from the L2 norm is proposed to solve the optimization problem of sparse signal reconstruction. This new algorithm integrates the characteristics of both convex optimization and non-convex optimization algorithm, making the algorithm approximate the global optimal sparse solution with higher probability and higher efficiency in the iterative process. The simulations are carried out on sparse signal reconstructions of one-dimensional signal and two-dimensional no sparse signal. Compared with the convex optimization algorithm of the minimum norm and the greedy algorithm of the matching tracking, the new proposed algorithm performs better in the precision and speed of reconstructing signal. Especially under the low sampling rate of sparse signal, the performance of L0GP is much outstanding. As a result, the required number of measurements for sparse signal of compressed sensing would be less and the speed of compressed sensing can be accelerated greatly.

## Figures and Tables

**Table 1:** The quantitative results of different algorithms.

Algorithm	SP	IRLS	OMP	ROMP	GOMP	L1_BP	L1_LS	L0GP
PSNR (dB)	20.08	22.04	21.67	21.18	21.89	21.87	22.03	22.40
SSIM	0.3440	0.5798	0.5141	0.4645	0.5697	0.5709	0.5794	0.6002
Time(s)	19.56	106.6	35.64	3.664	23.98	129.0	270.0	15.17

**Table 2:** The quantitative results (PSNR/SSIM) of different algorithms for the different types of images.

Algorithm	Peppers	Bridge	Lena	Barbara	Goldhill	Fingerprint
SP	23.37/0.5663	20.48/0.361	23.74/0.5661	21.99/0.5733	23.95/0.5243	19.31/0.3303
IRLS	27.71/0.7329	23.60/0.6513	28.86/0.7501	24.86/0.7774	28.22/0.7604	23.82/0.7620
OMP	27.58/0.7124	23.21/0.5818	28.60/0.7260	24.68/0.7462	27.60/0.7069	23.00/0.6905
ROMP	26.46/0.6839	22.67/0.5293	27.22/0.6928	23.60/0.6997	26.85/0.6761	21.87/0.6059
GOMP	27.69/0.7309	23.43/0.6467	28.83/0.7481	24.83/0.7751	28.02/0.7512	23.77/0.7588
L1_BP	27.68/0.7314	23.57/0.6489	28.83/0.7485	24.83/0.7757	28.04/0.7527	23.76/0.7600
L1_LS	27.89/0.7391	23.85/0.6624	29.18/0.7572	25.23/0.7899	28.40/0.7657	24.43/0.7816
L0GP	28.25/0.7521	24.32/0.6850	29.64/0.7692	25.59/0.8045	28.95/0.7839	24.98/0.7996

**Table 3:** The quantitative results of different arithmetics.

Algorithm	SP	IRLS	OMP	ROMP	GOMP	L1_BP	L1_LS	L0GP
PSNR (dB)	20.90	29.34	28.25	27.51	29.04	29.32	29.35	29.53
SSIM	0.5022	0.9296	0.9042	0.9096	0.9230	0.9293	0.9296	0.9310
Time(s)	67.63	290.5	266.3	15.40	61.20	276.9	300.3	40.71

**Table 4:** The quantitative results (PSNR/SSIM) of different algorithms for the different types of images.

Algorithm	Peppers	Bridge	Lena	Barbara	Goldhill	Mandrill
SP	26.07/0.6361	21.85/0.4598	26.70/0.6439	24.10/0.6830	25.89/0.6046	20.96/0.4385
IRLS	29.32/0.7860	25.86/0.8044	31.62/0.816	28.07/0.9084	30.46/0.8554	23.50/0.7396
OMP	29.28/0.7791	25.66/0.7810	31.53/0.8089	28.01/0.9005	30.27/0.8411	23.38/0.7165
ROMP	29.04/0.7709	25.19/0.7552	31.30/0.8001	27.63/0.8874	29.28/0.8212	22.98/0.6905
GOMP	29.30/0.7837	25.81/0.7983	31.58/0.8135	28.05/0.9062	30.40/0.8516	23.46/0.7330
L1_BP	29.31/0.785	25.85/0.8038	31.60/0.8150	28.06/0.9080	30.44/0.8547	23.48/0.7389
L1_LS	29.33/0.7866	25.89/0.8051	31.64/0.8164	28.09/0.9087	30.48/0.8559	23.53/0.7404
L0GP	29.43/0.7922	26.17/0.8145	31.90/0.8231	28.62/0.9152	30.78/0.8634	23.98/0.7551
